# Instability Is the Most Common Indication for Revision Hip Arthroplasty in the United States: National Trends From 2012 to 2018

**DOI:** 10.1016/j.artd.2021.08.001

**Published:** 2021-08-31

**Authors:** Alex Upfill-Brown, Peter P. Hsiue, Troy Sekimura, Jay N. Patel, Micah Adamson, Alexandra I. Stavrakis

**Affiliations:** Department of Orthopaedic Surgery, David Geffen School of Medicine at UCLA, Los Angeles, CA, USA

**Keywords:** Revision hip arthroplasty, Aseptic loosening, Prosthetic joint infection, Periprosthetic instability, Healthcare utilization

## Abstract

**Background:**

As primary total hip arthroplasty volume continues to increase, so will the number of revision total hip arthroplasty (rTHA) procedures. These complex cases represent a significant clinical and financial burden to the health-care system.

**Methods:**

This was a retrospective review using the National Inpatient Sample. International Classification of Diseases, 9th and 10^th^ revision codes were used to identify patients who underwent rTHA and create cohorts based on rTHA indications from 2012 to 2018. National and regional trends for length of stay (LOS), cost, and discharge location were evaluated.

**Results:**

A total of 292,250 rTHA procedures were identified. The annual number of rTHA procedures increased by 28.1% from 2012 to 2018 (37,325 to 47,810). The top 3 indications for rTHA were instability (20.4%), aseptic loosening (17.8%), and infection (11.1%). Over the study period, the proportion of patients discharged to skilled nursing facility decreased from 44.2% to 38% (*P* < .001). Hospital LOS decreased on average from 4.8 to 4.4 days (*P* < .001). Infections had the highest average LOS (7.3 days) followed by periprosthetic fractures (6.5 days). Hospital costs decreased over the study period, from $25,794 to $24,555 (*P* < .001). The proportion of rTHA cases performed at urban academic centers increased (58.0% to 75.3%, *P* < .001) while the proportion performed at urban nonacademic centers decreased (35.5% to 19.4%, *P* < .001).

**Conclusion:**

Instability was the most common indication for rTHA between 2012 and 2018. The proportion of rTHA performed in urban academic centers has increased substantially, away from urban nonacademic centers. While cost and LOS have decreased, significant geographic variability exists.

## Introduction

Total hip arthroplasty (THA) is tremendously effective in the treatment of severe, symptomatic arthritis, and is one of the most commonly performed surgical procedures in the United States [[1], [2], [3], [4], [5]]. Annual surgical volume of primary THA is growing and is projected to surpass 900,000 cases per year by 2030 [6,7]. Despite efforts to improve the durability and longevity of prosthetic constructs, the incidence of revision THA (rTHA) continues to rise and increased by 36% from 2002 to 2014 [6,8,9]. Owing to numerous factors including costlier implants and longer hospitalizations, the total financial burden associated with revision arthroplasty is significantly greater than that for primary arthroplasty, and on average, revision arthroplasty costs 76% more than primary joint replacement [[10], [11], [12], [13]]. Given the significant cost of revising failed primary THA, the economic implications of the projected increase in revision arthroplasty are tremendous.

Instability has previously been identified as the most common indication for rTHA, followed closely by aseptic loosening and periprosthetic joint infection (PJI) [10,11]. Numerous advancements over the last few decades have been implemented with the hopes of specifically reducing the rates of rTHA based on the aforementioned causes. For instance, the utilization of highly cross-linked polyethylene has been shown to decrease the risk of bearing surface wear and thereby reducing the incidence of rTHA for this indication [14].

Numerous advancements over the last few decades have been implemented with the hopes of specifically reducing rates of instability, ranging from modifications to surgical approach and soft-tissue repair; optimizing implant positioning via computer navigation or robotic assistance; maximizing prosthetic femoral head size; and modifying bearing surfaces such as through the use of dual mobility [[15], [16], [17], [18]]. Whether or not these advances have had any impact on decreasing the incidence of instability/dislocation after THA remains an important question.

The advent of large, nationally representative databases have given clinician-scientists access to tremendous amounts of data and the statistical power to investigate rare clinical events. The National Inpatient Sample (NIS), first made available in 1988, is one such database, which aggregates data from hospital discharges within the United States to allow analysis of health-care utilization, quality of care, and patient outcomes [19,20]. The NIS has been used extensively in many fields of medicine to better understand the delivery of care and patient outcomes [[21], [22], [23], [24]]. The NIS uses standardized coding protocols, including the International Classification of Diseases (ICD) to allow for efficient analysis of patient data. Throughout its history, the NIS has used various iterations of the ICD, and in the fourth quarter of 2015, it switched from the use of International Classification of Diseases, 9^th^ Revision, (ICD-9) to the newer International Classification of Diseases, 10^th^ Revision, (ICD-10) standard [20]. While the introduction ICD-10 has allowed providers to document patient progress with added granularity, the increased complexity of ICD-10 has concurrently presented challenges to consistently tracking patient outcomes during the transition from ICD-9 [[25], [26], [27]]. Since this change, there has been a paucity of data describing updated trends in revision arthroplasty.

Keeping abreast of the common indications for rTHA is of paramount importance for orthopedic surgeons, as it may help to direct further refinement of surgical techniques and prosthetic constructs to improve prosthesis longevity and patient outcomes and to minimize the need for revision surgery. The goal of this study was to provide an updated understanding of the incidence, indications for, and financial burden of rTHA in the United States.

## Material and methods

Our study cohort was identified using the NIS over a 6-year period (January 1, 2012, to December 31, 2018). The NIS is a nationally representative database developed from all hospitals participating in the Healthcare Cost and Utilization Project (HCUP) and validated through a federal-state-industry partnership sponsored by the Agency for Healthcare Research and Quality. It is based on inpatient data from over 40 states derived from billing and discharge information, covering approximately 96% of the U.S. population using an estimate of 20% stratified sample of discharges from U.S. hospitals. A stratified formula based on discharge weights reported by participating HCUP institutions was designed to allow an estimation of nationally representative statistics. Available variables include demographic data, diagnoses, procedures, hospital length of stay (LOS), hospital cost, and hospital characteristics [28]. As the NIS database has been sufficiently deidentified of any personal health information or identifiers, this study was deemed exempt by the institutional review board at our institution.

Patients older than 18 years who were admitted and underwent an rTHA procedure during the study period were considered for this study. Patients were identified using the ICD-9 as well as ICD-10 procedure codes for rTHA (Table 1). In order to be identified as a revision operation, each patient entry must contain either the revision code or both the removal and replacement codes. Patients with acetabular and femur revision procedure codes reported separately were classified as a revision of both components. These patients were then grouped based on the specific indication for rTHA (Supplementary Table 1). The first related diagnostic code listed was used as the primary indication for rTHA. The proportion of patients with multiple related diagnoses was summarized. For ICD-10 diagnostic codes, no differentiation was made between modifiers for initial encounter, subsequent encounter, or sequalae. The number of rTHA procedures per year was tabulated and used to generate the utilization trend during the study period.

**Table 1:** Procedural codes used to identify rTHA procedure types.

Location	Operation	ICD-9	ICD-10
Hip	Revision	00.70	0SW908Z, 0SW90EZ, 0SW90JZ, 0SWB08Z, 0SWB0EZ, 0SWB0JZ
	Removal		0SP908Z, 0SP90EZ, 0SP90JZ, 0SPB08Z, 0SPB0EZ, 0SPB0JZ
	Replacement		0SR9019, 0SR901A, 0SR901Z, 0SR9029, 0SR902A, 0SR902Z, 0SR9039, 0SR903A, 0SR903Z, 0SR9049, 0SR904A, 0SR904Z, 0SR9069, 0SR906A, 0SR906Z, 0SR90EZ, 0SR90J9, 0SR90JA, 0SR90JZ, 0SRB019, 0SRB01A, 0SRB01Z, 0SRB029, 0SRB02A, 0SRB02Z, 0SRB039, 0SRB03A, 0SRB03Z, 0SRB049, 0SRB04A, 0SRB04Z, 0SRB069, 0SRB06A, 0SRB06Z, 0SRB0EZ, 0SRB0J9, 0SRB0JA, 0SRB0JZ
Femur	Revision	00.72	0SWR0JZ, 0SWS0JZ
	Removal		0SPR0JZ, 0SPS0JZ, 0SP908Z, 0SP90EZ, 0SP90JZ, 0SPB08Z, 0SPB0EZ, 0SPB0JZ
	Replacement		0SRR019, 0SRR01A, 0SRR01Z, 0SRR039, 0SRR03A, 0SRR03Z, 0SRR0J9, 0SRR0JA, 0SRR0JZ, 0SRS019, 0SRS01A, 0SRS01Z, 0SRS039, 0SRS03A, 0SRS03Z, 0SRS0J9, 0SRS0JA, 0SRS0JZ
Acetabulum	Revision	00.71	0SWA0JZ, 0SWE0JZ
	Removal		0SPA0JZ, 0SPE0JZ, 0SP908Z, 0SP90EZ, 0SP90JZ, 0SPB08Z, 0SPB0EZ, 0SPB0JZ
	Replacement		0SRA009, 0SRA00A, 0SRA00Z, 0SRA019, 0SRA01A, 0SRA01Z, 0SRA039, 0SRA03A, 0SRA03Z, 0SRA0J9, 0SRA0JA, 0SRA0JZ, 0SRE009, 0SRE00A, 0SRE00Z, 0SRE019, 0SRE01A, 0SRE01Z, 0SRE039, 0SRE03A, 0SRE03Z, 0SRE0J9, 0SRE0JA, 0SRE0JZ

Patient demographics, hospital characteristics, hospitalization LOS, hospitalization cost, and discharge locations for patients undergoing rTHA were analyzed. Patient demographics included age (years), sex (male and female), race (white, black, Hispanic, Asian, Native American, and other), and insurance (Medicare, Medicaid, private, and self-pay). Discharge locations included home and skilled nursing facility (SNF). Hospital characteristics included hospital type (urban nonteaching, urban teaching, and rural), hospital size based on number of beds (large, medium, and small), and region (Northeast, Midwest, South, and West). Individual hospitalization cost was calculated using diagnosis-related group codes multiplied by hospital-specific cost-to-charge ratios provided by the Agency for Healthcare Research and Quality. HCUP indices of the diagnosis-related group were then used to account for differences in hospitalization severity [29]. The cost was subsequently standardized for inflation using rates from the United States Bureau of Labor Statistics and described in December 2018 U.S. dollars.

All result sample sizes represented national annual estimates, accounting for individual discharge-level weights from the NIS's stratified two-stage cluster design. Descriptive statistics were used to describe both baseline characteristics and outcome parameters within each comparison group. Continuous variables were reported using mean and standard error. Proportions were reported using mean and 95% confidence interval. Analysis was performed using a two-tailed Student’s t-test after ensuring normal distributions. For skewed, nonparametric distributions, continuous variables are presented as median (interquartile range) and analyzed using the Wilcoxon rank-sum test. Chi-squared tests were used for categorical analysis. Trend analysis was performed using univariate regression evaluating a linear relationship for year. Statistical significance was defined as *P* < .05. Statistical analyses were performed using R 3.6.0 (R Foundation for Statistical Computing, Vienna, Austria).

## Results

A total of 292,250 rTHA procedures were identified during our study period within the NIS. From 2012 to 2018, the number of rTHA procedures increased by 28.1% (37,325 to 47,810; Table 2). All component revisions were the most common rTHA procedure (57.1%), followed by femoral component only (29.2%) and acetabular component only (13.7%). The proportion of type of procedure varied over time with 58.1% of both component rTHAs in 2012 decreasing to 54.1% in 2018 (*P* < .001) (Table 2).

**Table 2:** Annual numbers of rTHA procedures by procedure type.

Component	2012	2013	2014	2015	2016	2017	2018	All
Both	21685 (58.1%)	22470 (56.9%)	22885 (57.8%)	24185 (60.4%)	25725 (59.7%)	24050 (53.5%)	25875 (54.1%)	166875 (57.1%)
Femur	7865 (21.1%)	9525 (24.1%)	9520 (24.1%)	10125 (25.3%)	13815 (32.1%)	16710 (37.2%)	17740 (37.1%)	85300 (29.2%)
Acetabulum	7775 (20.8%)	7500 (19%)	7175 (18.1%)	5710 (14.3%)	3530 (8.2%)	4190 (9.3%)	4195 (8.8%)	40075 (13.7%)
Total	37,325	39,495	39,580	40,020	43,070	44,950	47,810	292,250

### Demographics

Of patients undergoing rTHA, 30.5% were aged 75 years or older, while 15.5% of patients were younger than 55 years (Supplementary Table 2). The proportion of patients younger than 55 years decreased from 18.5% in 2012 to 12.9% in 2018 (*P* < .001). The proportion of patients aged 65 to 74 years undergoing rTHA increased from 27.1% to 31.9% (*P* < .001). Insurance type remained relatively stable over the study period with Medicare being the payer in 63.8% of patients, private insurance in 27.5%, Medicaid in 5.1%, and other means in 3.5% (Supplementary Table 3).

### Indications

**Figure 1:**
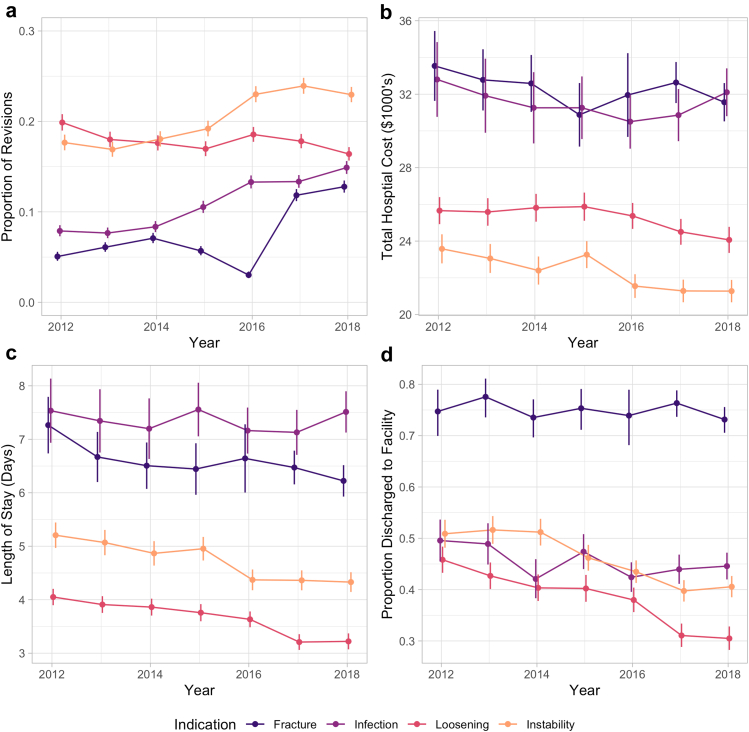
Trends in annual rTHA by primary associated indication for overall proportion (a), mean hospital costs (b), length of stay (c), and proportion discharge to facility (d). Vertical bars represent 95% confidence intervals.

The top 3 associated primary indications for rTHA were instability (20.4%), aseptic loosening (17.8%), and PJI (11.1%) (Table 3, Fig. 1a). Over time, the proportion of rTHA procedures associated with instability increased from 17.7% in 2012 to 23.0% in 2018 (*P* < .001). Similarly, the proportion of procedures associated with PJI increased from 7.9% in 2012 to 14.9% in 2018 (*P* < .001). The trend was opposite for aseptic loosening, with the proportion of rTHA attributed to this diagnosis decreasing from 19.9% in 2012 to 16.4% in 2018 (*P* < .001). Importantly, the proportion of cases with a nonspecific diagnostic code classified as “other” decreased from 29.2% in 2012 to 16.3% in 2018 (*P* < .001). A total of 13.4% of cases were not associated with any associated diagnosis codes, while 17.3% of patients had multiple diagnosis codes listed.

**Table 3:** Primary diagnosis for patients undergoing rTHA.

Diagnosis	2012	2013	2014	2015	2016	2017	2018	Total
Loosening	7420 (19.9%)	7110 (18%)	6970 (17.6%)	6790 (17%)	7990 (18.6%)	8005 (17.8%)	7840 (16.4%)	52125 (17.8%)
PJI	2945 (7.9%)	3025 (7.7%)	3305 (8.4%)	4215 (10.5%)	5725 (13.3%)	6000 (13.3%)	7120 (14.9%)	32335 (11.1%)
Instability	6590 (17.7%)	6675 (16.9%)	7145 (18.1%)	7685 (19.2%)	9905 (23%)	10755 (23.9%)	10975 (23%)	59730 (20.4%)
Bearing surface wear	1435 (3.8%)	1365 (3.5%)	1185 (3%)	1340 (3.3%)	1860 (4.3%)	1935 (4.3%)	1940 (4.1%)	11060 (3.8%)
Periprosthetic fracture	1890 (5.1%)	2405 (6.1%)	2805 (7.1%)	2275 (5.7%)	1300 (3%)	5320 (11.8%)	6110 (12.8%)	22105 (7.6%)
Osteolysis	1135 (3%)	955 (2.4%)	910 (2.3%)	880 (2.2%)	1085 (2.5%)	925 (2.1%)	965 (2%)	6855 (2.3%)
Breakage	735 (2%)	620 (1.6%)	535 (1.4%)	745 (1.9%)	980 (2.3%)	900 (2%)	895 (1.9%)	5410 (1.9%)
Other	10890 (29.2%)	12500 (31.6%)	11935 (30.2%)	10670 (26.7%)	7525 (17.5%)	7325 (16.3%)	7815 (16.3%)	68660 (23.5%)
Missing	4285 (11.5%)	4840 (12.3%)	4790 (12.1%)	5420 (13.5%)	6700 (15.6%)	3785 (8.4%)	4150 (8.7%)	33970 (11.6%)
Total	37,325	39,495	39,580	40,020	43,070	44,950	47,810	292,250

**Figure 2:**
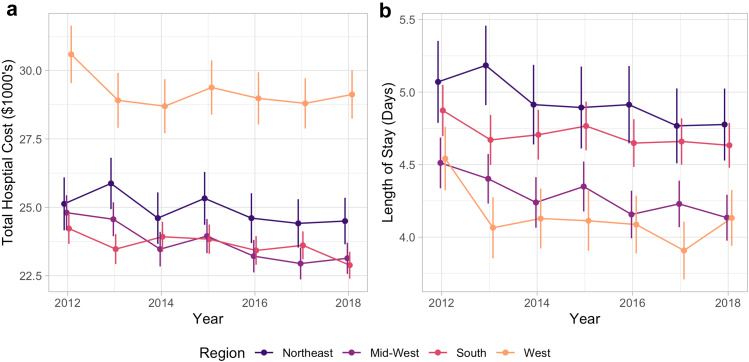
Total hospital costs (a) and length of stay (b) by US census region. Vertical bars represent 95% confidence interval. USD adjusted for inflation, represented as December 2018 USD.

### Cost

While average hospital charges increased significantly from $86,433 to $99,622 over the study period (*P* < .001; Supplementary Table 4), hospital costs decreased from $25,794 in $2012 to $24,555 in 2018 (*P* < .001; Table 4). Periprosthetic fractures ($32,204) had the highest average cost followed by PJI ($31,443). The average hospital cost associated with PJI (*P* = .532) and periprosthetic fracture (*P* = .141) did not increase significantly over the study period, while the mean hospital cost associated with instability (from $25,378 to 21,276, *P* < .001, Fig. 1b) and loosening (from $25,660 to 24,065, *P* < .001) decreased.

**Table 4:** Total hospital costs in USD, adjusted for inflation, by primary rTHA indication.

Diagnosis	2012	2013	2014	2015	2016	2017	2018	Total
Loosening	25660 (367)	25587 (358)	25816 (449)	25875 (422)	25371 (344)	24504 (340)	24065 (341)	25234 (141)
PJI	32802 (1242)	31916 (874)	31259 (875)	31261 (887)	30505 (650)	30861 (653)	32104 (776)	31443 (314)
Instability	23578 (471)	23055 (416)	22397 (360)	23262 (390)	21550 (320)	21286 (277)	21276 (318)	22160 (134)
Bearing surface wear	19634 (641)	19668 (721)	18009 (612)	18170 (544)	17579 (458)	16545 (438)	16556 (366)	17849 (200)
Periprosthetic fracture	33540 (1051)	32780 (748)	32586 (884)	30877 (740)	31954 (1091)	32635 (587)	31562 (531)	32204 (281)
Osteolysis	26178 (1044)	26686 (1423)	22718 (892)	24256 (856)	25008 (956)	22395 (923)	20387 (726)	24018 (384)
Breakage	27671 (1291)	28418 (1700)	27812 (1348)	28621 (1348)	26109 (1160)	27011 (1248)	24874 (1019)	27036 (488)
Other	22622 (284)	21910 (252)	21768 (257)	21938 (301)	20414 (283)	20236 (300)	19236 (280)	21348 (107)
Missing	30895 (777)	29597 (708)	28240 (482)	28239 (452)	28830 (466)	26518 (597)	25546 (540)	28355 (217)
Total	25794 (210)	25289 (184)	24935 (179)	25262 (185)	24727 (171)	24679 (171)	24555 (185)	25003 (69)

Standard error in parentheses.

### Length of stay

Hospital LOS decreased over the study period for all rTHAs from 4.75 to 4.43 days (*P* < .001; Table 5). PJI had the highest average LOS (7.34 days), followed by periprosthetic fractures (6.50 days). Over the study period, LOS decreased significantly for rTHA associated with instability (*P* < .001), aseptic loosening (*P* < .001), and periprosthetic fracture (*P* = .004; Fig. 1c). Change in LOS was not significant for PJI (*P* = .846).

**Table 5:** Mean length of stay by primary rTHA indication.

Diagnosis	2012	2013	2014	2015	2016	2017	2018	Total
Loosening	4.05 (0.08)	3.91 (0.07)	3.86 (0.09)	3.76 (0.09)	3.63 (0.07)	3.21 (0.07)	3.22 (0.07)	3.65 (0.03)
PJI	7.53 (0.31)	7.34 (0.3)	7.2 (0.26)	7.56 (0.26)	7.16 (0.21)	7.13 (0.22)	7.51 (0.21)	7.34 (0.09)
Instability	5.21 (0.14)	5.07 (0.11)	4.87 (0.1)	4.95 (0.13)	4.37 (0.09)	4.36 (0.09)	4.33 (0.09)	4.67 (0.04)
Bearing surface wear	3.25 (0.16)	3.16 (0.18)	2.63 (0.08)	2.94 (0.14)	2.5 (0.08)	2.35 (0.1)	2.07 (0.08)	2.65 (0.04)
Periprosthetic fracture	7.26 (0.45)	6.67 (0.21)	6.51 (0.19)	6.44 (0.21)	6.64 (0.31)	6.47 (0.17)	6.22 (0.13)	6.5 (0.08)
Osteolysis	3.87 (0.17)	3.77 (0.3)	3.41 (0.19)	3.02 (0.16)	3.11 (0.15)	2.8 (0.15)	2.61 (0.18)	3.24 (0.07)
Breakage	4.89 (0.3)	5.19 (0.46)	4.89 (0.36)	4.87 (0.3)	4.39 (0.22)	4.28 (0.29)	3.85 (0.23)	4.56 (0.11)
Other	3.67 (0.06)	3.51 (0.06)	3.45 (0.06)	3.21 (0.06)	3.01 (0.07)	2.93 (0.08)	2.71 (0.08)	3.27 (0.03)
Missing	5.66 (0.18)	5.28 (0.17)	5.12 (0.17)	5.03 (0.12)	5.18 (0.11)	4.28 (0.13)	3.99 (0.14)	4.98 (0.06)
Total	4.75 (0.06)	4.57 (0.05)	4.5 (0.05)	4.54 (0.05)	4.45 (0.05)	4.41 (0.05)	4.43 (0.05)	4.52 (0.02)

Standard error in parentheses.

### Discharge destination

Overall, the proportion of patients discharged to SNF decreased from 44.2% to 38% (*P* < .001) over the study period (Table 6). Patients treated for periprosthetic fractures were most likely to be discharged to SNF (73%). Over the study period, the proportion of patients discharged to SNF decreased significantly for those with aseptic loosening, instability, and PJI (*P* < .001 for all), while it did not change significantly for periprosthetic fracture (*P* = .06; Fig. 1d).

**Table 6:** Proportion of rTHA patients discharged to facility by primary indication.

Diagnosis	2012	2013	2014	2015	2016	2017	2018	Total
Loosening	45.1% (3345)	42.1% (2990)	40% (2790)	39.8% (2705)	37.7% (3010)	30.8% (2465)	30.3% (2375)	37.8% (19680)
PJI	48.2% (1420)	47.9% (1450)	41% (1355)	46.4% (1955)	41.7% (2390)	42.8% (2565)	43.6% (3105)	44% (14240)
Instability	50% (3295)	50.9% (3395)	50.7% (3620)	45.4% (3490)	43% (4255)	39.1% (4205)	40% (4385)	44.6% (26645)
Bearing surface wear	23.7% (340)	26.7% (365)	24.5% (290)	24.3% (325)	19.1% (355)	17.3% (335)	17.5% (340)	21.2% (2350)
Periprosthetic fracture	70.4% (1330)	75.5% (1815)	71.3% (2000)	74.5% (1695)	71.9% (935)	74.9% (3985)	71.8% (4385)	73% (16145)
Osteolysis	38.3% (435)	31.9% (305)	29.7% (270)	30.1% (265)	30.9% (335)	22.7% (210)	19.7% (190)	29.3% (2010)
Breakage	49.7% (365)	46% (285)	40.2% (215)	50.3% (375)	43.9% (430)	37.8% (340)	35.2% (315)	43% (2325)
Other	34.7% (3780)	31.2% (3905)	31.8% (3790)	28% (2990)	26.1% (1965)	22.7% (1665)	21.8% (1700)	28.8% (19795)
Missing	51.1% (2190)	53.6% (2595)	46.8% (2240)	50.6% (2740)	54.5% (3650)	37.3% (1410)	33.4% (1385)	47.7% (16210)
Total	44.2% (16500)	43.3% (17105)	41.9% (16570)	41.3% (16540)	40.2% (17325)	38.2% (17180)	38% (18180)	40.9% (119400)

### Regional analyses

The distribution of rTHA cases by census region was constant over the study period, with the highest proportion of cases in the Southern region (36.1%), followed by the Midwest (24.7%), the West (21.4%), and the Northeast (17.8%) (Supplementary Table 5). rTHA Procedures performed in the West were associated with the lowest average LOS of 4.13 days but the highest average cost of $29,179 (Supplementary Table 6, Supplementary Table 7). The mean total hospital costs decreased significantly in the South (*P* = .002), Northeast (*P* = .048), and Midwest (<0.001), but costs did not change significantly in the West (*P* = .14, Fig. 2). Meanwhile, the mean LOS decreased significantly in the West (*P* = .008), Northeast (*P* = .017), and Midwest (*P* < .001), but not the South (*P* = .08, Fig. 2).

### Hospital type analyses

**Figure 3:**
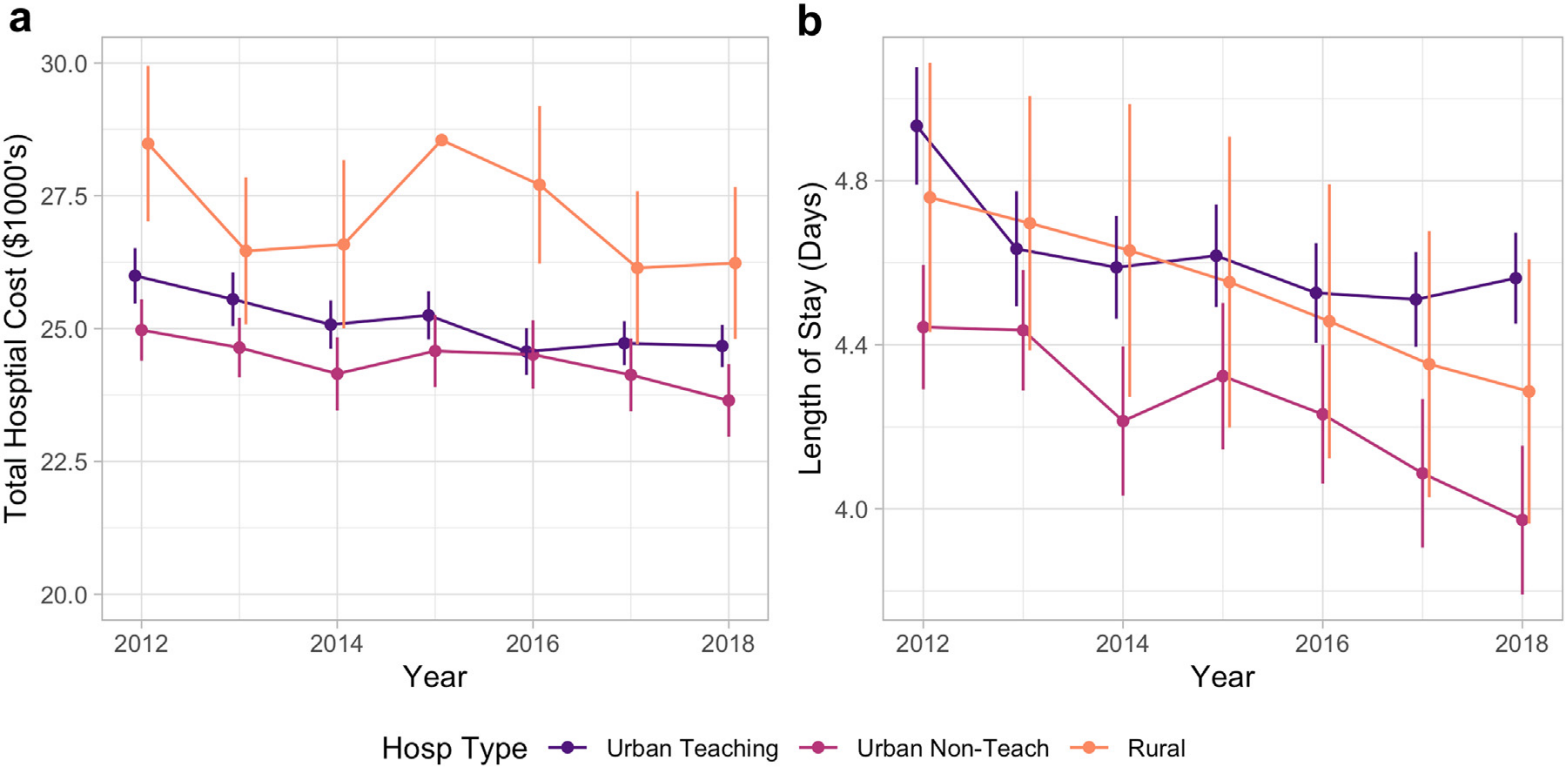
Total hospital costs (a) and length of stay (b) by hospital type. Vertical bars represent 95% confidence interval. USD adjusted for inflation, represented as December 2018 USD.

The proportion of rTHA cases performed at urban academic centers significantly increased from 58.0% in 2012 to 75.3% in 2018 (*P* < .001), while the proportion performed at urban nonacademic centers (35.5% to 19.4%, *P* < .001) and rural centers (6.5% to 5.3%, *P* < .001) decreased over the study period (Table 7). Hospital costs decreased for urban teaching (*P* < .001) and nonteaching (*P* = .006) hospitals, but not in rural hospitals (*P* = .11) over the study period (Supplementary Table 8, Fig. 3). Compared to urban teaching hospitals, costs were lower at urban nonteaching hospitals (*P* < .001) and higher in rural hospitals (*P* < .001). LOS decreased significantly in all hospital types (Supplementary Table 9) and was significantly shorter in urban nonteaching (*P* < .001) but not rural hospitals (*P* = .36) than in teaching hospitals. The indications for rTHA by hospital type are summarized in Supplementary Table 10. Overall, there was no difference in the proportion of rTHA attributable to aseptic loosening by hospital type (*P* = .60). Rural hospitals had the highest rate of rTHA for instability (*P* < .001) and periprosthetic fracture (*P* = .04), while urban academic centers had the highest rate of rTHA for PJI (*P* < .001).

**Table 7:** Number of rTHA cases by hospital type.

Hospital type	2012	2013	2014	2015	2016	2017	2018	Total
Urban teaching	21650 (58%)	22700 (57.5%)	28280 (71.5%)	28360 (70.9%)	30080 (69.8%)	33200 (73.9%)	36010 (75.3%)	200280 (68.5%)
Urban nonteaching	13245 (35.5%)	14075 (35.6%)	9245 (23.4%)	9580 (23.9%)	10650 (24.7%)	9270 (20.6%)	9285 (19.4%)	75350 (25.8%)
Rural	2430 (6.5%)	2720 (6.9%)	2055 (5.2%)	2080 (5.2%)	2340 (5.4%)	2480 (5.5%)	2515 (5.3%)	16620 (5.7%)
Total	37,325	39,495	39,580	40,020	43,070	44,950	47,810	292,250

## Discussion

THA is one of the most commonly performed procedures in the United States and is associated with excellent outcomes. Sloan predicted the number of THA procedures performed annually in the U.S. to reach 1.23 million by the year 2060 [30]. During this same time period, Sloan also predicts the number of rTHA performed to increase by 219% to 110,000 annually. With the increasing number of rTHA, it becomes increasingly important to study the paradigms and shifts in various trends to evaluate their efficacy and financial prudence as they relate to rTHA. In addition, analysis of the causes and patterns associated with rTHA can assess current systems and guide future research. While a large nationally representative database such as NIS allows for such an analysis, conclusions must be tempered because of the reliance on reported ICD codes and their associated inaccuracies.

The proportion of rTHA performed due to aseptic loosening has decreased significantly over the study period from 19.9% in 2012 to 16.4% in 2018. The primary driver for this trend is likely the adoption of highly cross-linked polyethylene in acetabular liners, which has been increasing since the early 2000s [[31], [32], [33], [34], [35], [36]]. Multiple randomized control studies and registry studies have repeatedly demonstrated that highly cross-linked polyethylene liners have lower rates of osteolysis and subsequent revision for aseptic loosening than conventional ultra-high-molecular-weight polyethylene liners [31,33,34,36,37]. Prior studies of rTHA epidemiology have not examined annual trends, but for the periods of 2005 – 2010 and 2009 – 2013, aseptic loosening accounted for 20% and 16.8% of all rTHA procedures, respectively [10,38]. These studies included isolated liner exchanges and removal of prostheses — both with associated lower rates of aseptic loosening — which may have led to lower estimated rates of aseptic loosening relative to this study [10]. Overall, the decreasing rates of rTHA performed for aseptic loosening over the past 15 years is likely a reflection of the well-studied effect of the use of highly cross-linked polyethylene liners.

On the other hand, the proportion of rTHA procedures associated with instability increased from 17.7% in 2012 to 23.0% in 2018. This finding is of particular interest as there have been many advances to attempt to mitigate this complication: the focus on re-establishing hip length, offset, combined anteversion, the popularization of dual mobility liners, and the increased use of the direct anterior approach [[39], [40], [41]]. Robot-assisted THA, which has been shown to decrease component positioning outliers, has also been developed in part to decrease instability and dislocation risk [[42], [43], [44]]. Despite all these and other measures, the incidence of THA instability continues to rise. Previous studies also examining rTHA trends between 2005 – 2010 and 2009 – 2013 have likewise noted instability to be the most common cause of rTHA [10,38]. Although this problem has been identified and various solutions have been developed, the continued growth of instability is problematic. THA stability is multifactorial and is more complex than simply acetabular component placement within the Lewinnek safe zone [45]. Further research is needed studying the intricacies and additional contributing factors associated with hip stability such as the spinopelvic relationship [46].

With the evolution of rapid recovery protocols, advances in regional anesthesia, and the added weight of bundled care payment models, LOS and discharge destination after surgery has evolved. Over the study period, LOS decreased for all the major rTHA indications but was particularly significant for patients treated for instability and aseptic loosening. PJIs had the highest average LOS (7.34 days) followed by periprosthetic fractures (6.50 days). This can be partially due to finalization of intraoperative cultures and coordination of long-term intravenous antibiotics for patients being treated for PJIs. Decreased mobility and limited weight-bearing after periprosthetic fracture rTHA may play a role in hindering patients’ ability to be cleared for a safe discharge home, requiring additional time in the hospital as well as additional support after discharge. This may also be why there was a decrease in discharge to an SNF over the study period across all rTHA indications with the exception of periprosthetic fractures.

Despite increasing hospital charges for rTHA, hospital costs actually decreased from 2012 to 2018 — consistent with the shorter LOS observed over this period. However, costs did not decrease significantly in the Western census region or in rural hospitals. Periprosthetic fractures ($32,204) had the highest average cost followed by PJIs ($31,443), and neither changed significantly over the study period. Phillips et al. analyzed the cost of readmission for revision total joint arthroplasty under the bundled payment model and found significantly higher rates of postacute care and overall episode of care costs than readmission for medical complications [47]. The cost associated with rTHA was also variable among the different regions of the United States. Specifically, rTHA procedures performed in the West of the United States were associated with the lowest average LOS of 4.13 days; however, they had the highest average cost, $29,179. Also, the West was the only region in which hospital costs did not decrease significantly. Similarly, while urban teaching and nonteaching hospitals saw significant decreases in costs for rTHA, rural hospitals did not. Further studies are needed to analyze the geographic disparities in LOS and costs for comparable procedures performed in other regions of the United States.

rTHA Are often complex requiring additional training, surgical expertise, and multidisciplinary care which may be more commonly found at academic institutions. Kowalik et al. explored the epidemiology of rTHA between teaching and nonteaching hospitals in the U.S. from 2006 to 2010 [48]. In their study, 54% of all rTHA procedures were performed at teaching hospitals. The proportion of rTHAs performed at teaching hospitals has continued to grow with 75.3% of rTHA cases being performed at academic institutions in 2018, in the present study. Based on our data, urban nonacademic centers saw a drop in the rate of rTHA from 35.5% to 19.4%. The proportion of patients undergoing rTHA at rural centers also decreased between 2012 and 2018.

The present study is not without its limitations. First, we recognize the inherent weaknesses in a large database study including potential for errors in coding and data entry. The transition to using ICD-10 codes in October 2015 was likely associated with increased variations in coding as new norms were being established [49]. However, our study is one of the first to use ICD-10 codes in a database study evaluating rTHA. We hope that future studies can continue to clarify and improve upon the procedure and diagnostic codes used to accurately capture and evaluate these patients. Given the limited granularity of ICD codes and coding errors, it is possible that types of conversion THA, such as conversion of hemiarthroplasty or hip-resurfacing arthroplasty, are included in our analysis of rTHA. This study did not evaluate any outcomes after the initial rTHA admission because the NIS does not include readmission data. Given the increased risk of complications after rTHA, it would be useful to evaluate how outcomes after rTHA have changed over the last decade. Finally, information regarding surgical details such as implants used, procedure duration, intraoperative complications, and blood loss was unavailable in the NIS. Thus, we were unable to comment on changes in these variables over time.

## Conclusions

Despite these limitations, our study, to the best of our knowledge, reports on the largest number of patients undergoing rTHA to date and provides the most recent national epidemiological analysis. Our findings highlight some of the most recent trends in rTHA which will be important to consider as the number of rTHA procedures is projected to increase in the coming years.

## Conflicts of interest

The authors declare that they have no known competing financial interests or personal relationships that could have appeared to influence the work reported in this article.

## Figures and Tables

**Supplementary Table 1:** Categorized rTHA diagnostic codes for ICD-9 and ICD-10.

Group	ICD-9	ICD-10	ICD10 Text
Loosening	996.41	T84.03	Mechanical loosening of internal prosthetic joint
		T84.030	Mechanical loosening of internal right hip prosthetic joint
		T84.031	Mechanical loosening of internal left hip prosthetic joint
		T84.038	Mechanical loosening of other internal prosthetic joint
		T84.039	Mechanical loosening of unspecified internal prosthetic joint
Infection	996.66	T84.5	Infection and inflammatory reaction due to internal joint prosthesis
		T84.50	Infection and inflammatory reaction due to unspecified internal joint prosthesis
		T84.51	Infection and inflammatory reaction due to internal right hip prosthesis
		T84.52	Infection and inflammatory reaction due to internal left hip prosthesis
		T84.59	Infection and inflammatory reaction due to other internal joint prosthesis
	996.69	T84.7	Infection and inflammatory reaction due to other internal orthopedic prosthetic devices, implants and grafts
Instability	996.42	T84.02	Dislocation of internal joint prosthesis
		T84.020	Dislocation of internal right hip prosthesis
		T84.021	Dislocation of internal left hip prosthesis
		T84.028	Dislocation of other internal joint prosthesis
		T84.029	Dislocation of unspecified internal joint prosthesis
		M24.3	Pathological dislocation of joint, not elsewhere classified
		M24.35	Pathological dislocation of hip, not elsewhere classified
		M24.4	Recurrent dislocation of joint
		M24.45	Recurrent dislocation, hip
		S73.0	Subluxation and dislocation of hip
		S73.00	Unspecified subluxation and dislocation of hip
		S73.001	Unspecified subluxation of right hip
		S73.002	Unspecified subluxation of left hip
		S73.003	Unspecified subluxation of unspecified hip
		S73.004	Unspecified dislocation of right hip
		S73.005	Unspecified dislocation of left hip
		S73.006	Unspecified dislocation of unspecified hip
		S73.01	Posterior subluxation and dislocation of hip
		S73.011	Posterior subluxation of right hip
		S73.012	Posterior subluxation of left hip
		S73.013	Posterior subluxation of unspecified hip
		S73.014	Posterior dislocation of right hip
		S73.015	Posterior dislocation of left hip
		S73.016	Posterior dislocation of unspecified hip
		S73.02	Obturator subluxation and dislocation of hip
		S73.021	Obturator subluxation of right hip
		S73.022	Obturator subluxation of left hip
		S73.023	Obturator subluxation of unspecified hip
		S73.024	Obturator dislocation of right hip
		S73.025	Obturator dislocation of left hip
		S73.026	Obturator dislocation of unspecified hip
		S73.03	Other anterior subluxation and dislocation of hip
		S73.031	Other anterior subluxation of right hip
		S73.032	Other anterior subluxation of left hip
		S73.033	Other anterior subluxation of unspecified hip
		S73.034	Other anterior dislocation of right hip
		S73.035	Other anterior dislocation of left hip
		S73.036	Other anterior dislocation of unspecified hip
		S73.04	Central subluxation and dislocation of hip
		S73.041	Central subluxation of right hip
		S73.042	Central subluxation of left hip
		S73.043	Central subluxation of unspecified hip
		S73.044	Central dislocation of right hip
		S73.045	Central dislocation of left hip
		S73.046	Central dislocation of unspecified hip
Breakage	996.43	T84.01	Broken internal joint prosthesis
		T84.010	Broken internal right hip prosthesis
		T84.011	Broken internal left hip prosthesis
		T84.018	Broken internal joint prosthesis, other site
		T84.019	Broken internal joint prosthesis, unspecified site
Periprosthetic fracture	996.44	M97	Periprosthetic fracture around internal prosthetic joint
		M97.0	Periprosthetic fracture around internal prosthetic hip joint
		M97.01	Periprosthetic fracture around internal prosthetic right hip joint
		M97.02	Periprosthetic fracture around internal prosthetic left hip joint
		M97.8	Periprosthetic fracture around other internal prosthetic joint
		M97.9	Periprosthetic fracture around unspecified internal prosthetic joint
Osteolysis	996.45	T84.05	Periprosthetic osteolysis of internal prosthetic joint
		T84.050	Periprosthetic osteolysis of internal prosthetic right hip joint
		T84.051	Periprosthetic osteolysis of internal prosthetic left hip joint
		T84.058	Periprosthetic osteolysis of other internal prosthetic joint
		T84.059	Periprosthetic osteolysis of unspecified internal prosthetic joint
		M89.5	Osteolysis
		M89.55	Osteolysis, thigh
Bearing surface wear	996.46	T84.06	Wear of articular bearing surface of internal prosthetic joint
		T84.060	Wear of articular bearing surface of internal prosthetic right hip joint
		T84.061	Wear of articular bearing surface of internal prosthetic left hip joint
		T84.068	Wear of articular bearing surface of other internal prosthetic joint
		T84.069	Wear of articular bearing surface of unspecified internal prosthetic joint
Other mechanical	996.47	T84.09	Other mechanical complication of internal joint prosthesis
		T84.090	Other mechanical complication of internal right hip prosthesis
		T84.091	Other mechanical complication of internal left hip prosthesis
		T84.098	Other mechanical complication of other internal joint prosthesis
		T84.099	Other mechanical complication of unspecified internal joint prosthesis
	996.49	T84.4	Mechanical complication of other internal orthopedic devices, implants, and grafts
		T84.41	Breakdown (mechanical) of other internal orthopedic devices, implants and grafts
		T84.418	Breakdown (mechanical) of other internal orthopedic devices, implants and grafts
		T84.42	Displacement of other internal orthopedic devices, implants and grafts
		T84.428	Displacement of other internal orthopedic devices, implants and grafts
		T84.49	Other mechanical complication of other internal orthopedic devices, implants and grafts
		T84.498	Other mechanical complication of other internal orthopedic devices, implants and grafts
		T84.3	Mechanical complication of other bone devices, implants and grafts
		T84.31	Breakdown (mechanical) of other bone devices, implants and grafts
		T84.318	Breakdown (mechanical) of other bone devices, implants and grafts
		T84.32	Displacement of other bone devices, implants and grafts
		T84.328	Displacement of other bone devices, implants and grafts
		T84.39	Other mechanical complication of other bone devices, implants and grafts
		T84.398	Other mechanical complication of other bone devices, implants and grafts
Other	996.77	T84.8	Other specified complications of internal orthopedic prosthetic devices, implants and grafts
		T84.81	Embolism due to internal orthopedic prosthetic devices, implants and grafts
		T84.82	Fibrosis due to internal orthopedic prosthetic devices, implants and grafts
		T84.83	Hemorrhage due to internal orthopedic prosthetic devices, implants and grafts
		T84.84	Pain due to internal orthopedic prosthetic devices, implants and grafts
		T84.85	Stenosis due to internal orthopedic prosthetic devices, implants and grafts
		T84.86	Thrombosis due to internal orthopedic prosthetic devices, implants and grafts
		T84.89	Other specified complication of internal orthopedic prosthetic devices, implants and grafts
		T84.9	Unspecified complication of internal orthopedic prosthetic device, implant and graft

**Supplementary Table 2:** Age groups of patients undergoing rTHA.

Age	2012	2013	2014	2015	2016	2017	2018	All
<55	6910 (18.5%)	6755 (17.1%)	6470 (16.3%)	6355 (15.9%)	6275 (14.6%)	6315 (14%)	6160 (12.9%)	45240 (15.5%)
55-64	8675 (23.2%)	9995 (25.3%)	9890 (25%)	10375 (25.9%)	10585 (24.6%)	10965 (24.4%)	11545 (24.1%)	72030 (24.6%)
65-74	10125 (27.1%)	10905 (27.6%)	11475 (29%)	11150 (27.9%)	12965 (30.1%)	14050 (31.3%)	15240 (31.9%)	85910 (29.4%)
75+	11615 (31.1%)	11840 (30%)	11735 (29.6%)	12125 (30.3%)	13245 (30.8%)	13620 (30.3%)	14860 (31.1%)	89040 (30.5%)
Total	37,325	39,495	39,580	40,020	43,070	44,950	47,810	292,250

**Supplementary Table 3:** Payor for patients undergoing rTHA.

Payers	2012	2013	2014	2015	2016	2017	2018	All
Medicare	23630 (63.3%)	24125 (61.1%)	24935 (63%)	24825 (62%)	28055 (65.1%)	29210 (65%)	31550 (66%)	186330 (63.3%)
Private	10635 (28.5%)	12090 (30.6%)	11455 (28.9%)	11550 (28.9%)	11265 (26.2%)	11495 (25.6%)	11930 (25%)	80420 (28.5%)
Medicaid	1545 (4.1%)	1585 (4%)	1915 (4.8%)	2145 (5.4%)	2395 (5.6%)	2610 (5.8%)	2730 (5.7%)	14925 (4.1%)
Other	1470 (3.9%)	1545 (3.9%)	1180 (3%)	1445 (3.6%)	1335 (3.1%)	1580 (3.5%)	1560 (3.3%)	10115 (3.9%)
Total	37,325	39,495	39,580	40,020	43,070	44,950	47,810	292,250

**Supplementary Table 4:** Average total hospital charges by rTHA indication.

Diagnosis	2012	2013	2014	2015	2016	2017	2018	Total
Loosening	86433 (1394)	92266 (1573)	94844 (1970)	95820 (1808)	97554 (1596)	99092 (1699)	99622 (1764)	95231 (641)
PJI	115397 (3829)	115958 (3639)	119593 (3787)	123542 (3634)	121999 (3201)	124974 (3540)	135651 (3566)	124397 (1413)
Instability	83330 (1811)	83397 (1637)	85173 (1703)	89834 (1837)	85508 (1602)	87461 (1362)	92467 (1574)	87200 (622)
Bearing surface wear	64603 (2581)	69407 (2714)	63351 (2734)	67560 (2386)	68029 (2220)	63773 (2228)	64653 (1852)	65845 (890)
Periprosthetic fracture	122656 (4651)	116509 (3299)	121882 (4245)	118524 (3674)	129782 (6052)	134544 (3277)	132812 (2663)	127602 (1423)
Osteolysis	88113 (4212)	95659 (4394)	79856 (3832)	83858 (3839)	92781 (4089)	89742 (4961)	85502 (3771)	88163 (1592)
Breakage	96054 (5012)	101925 (7163)	108687 (6594)	119193 (9023)	105070 (5432)	108861 (5643)	106630 (6023)	106758 (2454)
Other	78449 (1097)	79514 (1120)	82008 (1159)	84484 (1328)	80739 (1386)	81465 (1503)	80517 (1622)	81004 (488)
Missing	107453 (3784)	106272 (2424)	103884 (2160)	110162 (2377)	116620 (2290)	106055 (2599)	107095 (2518)	108828 (984)
Total	89490 (827)	91227 (739)	93230 (804)	97061 (842)	97848 (832)	100138 (868)	103539 (895)	96470 (324)

Standard error in parentheses.

**Supplementary Table 5:** Number of rTHA operations by census region.

Region	2012	2013	2014	2015	2016	2017	2018	All
Northeast	6730 (18%)	7145 (18.1%)	7120 (18%)	6735 (16.8%)	7555 (17.5%)	8060 (17.9%)	8705 (18.2%)	52050 (17.8%)
Midwest	9455 (25.3%)	9900 (25.1%)	9550 (24.1%)	9770 (24.4%)	10770 (25%)	11230 (25%)	11550 (24.2%)	72225 (24.7%)
South	13360 (35.8%)	14065 (35.6%)	14195 (35.9%)	14800 (37%)	15370 (35.7%)	16275 (36.2%)	17510 (36.6%)	105575 (36.1%)
West	7780 (20.8%)	8385 (21.2%)	8715 (22%)	8715 (21.8%)	9375 (21.8%)	9385 (20.9%)	10045 (21%)	62400 (21.4%)
Total	37,325	39,495	39,580	40,020	43,070	44,950	47,810	292,250

**Supplementary Table 6:** Average total hospital costs by US census region.

Region	2012	2013	2014	2015	2016	2017	2018	Total
Midwest	24805 (345)	24563 (328)	23471 (294)	23948 (360)	23215 (290)	22945 (280)	23137 (281)	23683 (117)
Northeast	25128 (458)	25873 (555)	24605 (429)	25327 (481)	24605 (393)	24413 (434)	24498 (503)	24893 (177)
South	24226 (327)	23474 (263)	23924 (288)	23836 (260)	23423 (265)	23610 (266)	22885 (231)	23596 (102)
West	30591 (603)	28911 (419)	28697 (463)	29382 (476)	28982 (466)	28800 (437)	29125 (549)	29179 (186)
Total	25794 (210)	25289 (184)	24935 (179)	25262 (185)	24727 (171)	24679 (171)	24555 (185)	25003 (69)

Standard error in parentheses.

**Supplementary Table 7:** Average length of stay for rTHA patients by US census region.

Region	2012	2013	2014	2015	2016	2017	2018	Total
Midwest	4.51 (0.09)	4.4 (0.09)	4.24 (0.08)	4.35 (0.1)	4.16 (0.08)	4.23 (0.09)	4.13 (0.08)	4.28 (0.03)
Northeast	5.07 (0.16)	5.18 (0.15)	4.91 (0.12)	4.89 (0.14)	4.91 (0.12)	4.77 (0.14)	4.78 (0.13)	4.92 (0.05)
South	4.87 (0.09)	4.67 (0.08)	4.71 (0.09)	4.77 (0.08)	4.65 (0.09)	4.66 (0.08)	4.63 (0.08)	4.7 (0.03)
West	4.54 (0.13)	4.06 (0.08)	4.13 (0.1)	4.11 (0.1)	4.09 (0.1)	3.91 (0.09)	4.13 (0.12)	4.13 (0.04)
Total	4.75 (0.06)	4.57 (0.05)	4.5 (0.05)	4.54 (0.05)	4.45 (0.05)	4.41 (0.05)	4.43 (0.05)	4.52 (0.02)

Standard error in parentheses.

**Supplementary Table 8:** Average hospital costs for rTHA treatment by hospital type.

Hospital type	2012	2013	2014	2015	2016	2017	2018	Total
Urban teaching	25995 (305)	25552 (258)	25075 (217)	25249 (224)	24566 (206)	24724 (201)	24672 (222)	25043 (87)
Urban nonteaching	24972 (286)	24641 (281)	24148 (337)	24578 (355)	24510 (336)	24127 (363)	23649 (350)	24424 (123)
Rural	28480 (755)	26462 (655)	26585 (859)	28547 (847)	27706 (758)	26141 (687)	26234 (743)	27125 (285)
Total	25794 (210)	25289 (184)	24935 (179)	25262 (185)	24727 (171)	24679 (171)	24555 (185)	25003 (69)

Standard error in parentheses.

**Supplementary Table 9:** Average length of stay for rTHA patients by hospital type.

Hospital type	2012	2013	2014	2015	2016	2017	2018	Total
Urban teaching	4.93 (0.08)	4.63 (0.07)	4.59 (0.06)	4.62 (0.06)	4.53 (0.06)	4.51 (0.06)	4.56 (0.06)	4.61 (0.02)
Urban nonteaching	4.44 (0.08)	4.44 (0.08)	4.21 (0.09)	4.32 (0.09)	4.23 (0.1)	4.09 (0.09)	3.97 (0.08)	4.27 (0.03)
Rural	4.76 (0.16)	4.7 (0.15)	4.63 (0.21)	4.55 (0.18)	4.46 (0.16)	4.35 (0.16)	4.29 (0.17)	4.53 (0.06)
Total	4.75 (0.06)	4.57 (0.05)	4.5 (0.05)	4.54 (0.05)	4.45 (0.05)	4.41 (0.05)	4.43 (0.05)	4.52 (0.02)

Standard error in parentheses.

**Supplementary Table 10:** Primary revision indication by hospital type.

Indication	Urban teaching	Urban nonteaching	Rural	Total
Loosening	35850 (17.9%)	13415 (17.8%)	2860 (17.2%)	52125 (17.8%)
PJI	23215 (11.6%)	7450 (9.9%)	1670 (10%)	32335 (11.1%)
Instability	39480 (19.7%)	16135 (21.4%)	4115 (24.8%)	59730 (20.4%)
Bearing surface wear	7710 (3.8%)	2795 (3.7%)	555 (3.3%)	11060 (3.8%)
Periprosthetic fracture	15130 (7.6%)	5540 (7.4%)	1435 (8.6%)	22105 (7.6%)
Osteolysis	5020 (2.5%)	1570 (2.1%)	265 (1.6%)	6855 (2.3%)
Breakage	3715 (1.9%)	1420 (1.9%)	275 (1.7%)	5410 (1.9%)
Other	46630 (23.3%)	18665 (24.8%)	3365 (20.2%)	68660 (23.5%)
Missing	23530 (11.7%)	8360 (11.1%)	2080 (12.5%)	33970 (11.6%)
Total	200,280	75,350	16,620	292,250

## Appendix A Supplementary data

Conflict of Interest for All Authors
